# Supplementary material for: Patterns of Intron Gain and Loss in Fungi
Source: PLoS Biol. 2004 Nov 30;2(12):e422. doi: 10.1371/journal.pbio.0020422 (PMC532390; doi:10.1371/journal.pbio.0020422)
Supplement: Table S1 — Also available at http://genes.mit.edu/NielsenEtAl/. (4.3 MB ZIP). [file pbio.0020422.st001.zip › NielsenEtAl/html/582.html]

AN0444.1.NCU06756.1.MG01115.1.FG06272.1


```
 CLUSTAL W (1.82) Multiple Sequence Alignments - Introns Inserted


Sequence 1: NCU06756.1	1322 aa
Sequence 2: FG06272.1	1188 aa
Sequence 3: MG01115.1	1365 aa
Sequence 4: AN0444.1	1165 aa
Alignment Length: 1408 aa
Number Identitical Residues: 343 aa
Alignment Score (without introns) 19098


MG01115.1 	MTTQQEQPSRLGGSTGVHHHRLDFRLPTTNTATSPPQWQDSSGLPHEEQQPPQPQLPVWQ
NCU06756.1	--MAIYTPQARSTREGTMNINDTSTPPSMAVSTTSPFWPLGPFPNRVDSHANSHPLPHQL
FG06272.1 	---------------------------------------MAPWLSRRGSQPNNS------
AN0444.1  	------------------------------------------------------------
          	                                                            

MG01115.1 	HHYHYHNQQNQNQQQQQLQYLHQKPLPPPPPPSHSPPPPPPSLSAPQTRQYRQPVPSPNR
NCU06756.1	QHPPQDPLQHPQAPFSQGSSQRQQQQQHPQWHPHPFPQSHRAFGPPPSAGSIITTNNLNR
FG06272.1 	------------------------------------------------------------
AN0444.1  	------------------------------------------------------------
          	                                                            

MG01115.1 	PRSPLRQRTFPNQASNAQTLPPARHLHHRQTSGSSLSNRGRPPPSNPGPCAVELWNRGVP
NCU06756.1	KYSNGNNNNLNGNSPGSGNLSSNVSVNRPPTANVQVRRR-PHAATTAGRFRSATRVPDLP
FG06272.1 	--SNLSNSDLITR-------IHNTALHTPTTLPALYSNN-LQAASNGNRF-------ELS
AN0444.1  	--------------------MPSLAAHKPSAHQSASSAG-VQPGTSPIPVP---------
          	                          :   :           . :.              

MG01115.1 	SPHQQQQYNPSTPSPRMTEFEFFDGSQLAESSDSDSDFVPKQAPQPRGHVRSMSHPFPSL
NCU06756.1	VLNNLRITSNTGNTN-------VDDSSSSDEGFSSPAARPQRPE----HRRSMSHPFPSL
FG06272.1 	FSN-----NNNGDSS-------SDESDLHPSRPSS--SRPQRPP----HTRSMSQPFPSL
AN0444.1  	--------IVGGRSP-------NRLRYGEPRVTGAPISPPSGPTFHRGHSRSISQPFASA
          	             :                   . .   *. .    .* **:*:**.* 

MG01115.1 	FSSNKKKKRSPAPRAYFDSRSSSEDSRDGSGLMPTASKGGAAPRISPQKQSLSGGASKD-
NCU06756.1	FSSKKKKAYQ------MYAGDSGSETVDDAGYAPKLGTSRPSTQHSRNHRNVPSTGSKD-
FG06272.1 	FSSKKKRQNS------VGAPPPDLGFADDDVVMPRQAP-KTHAQTPSHNGNGP-AGNKD-
AN0444.1  	LSGVGKRRNK------SISVEADVDTDDDDEDDDDIYIPNPNPISSSPRKGLPRVPPKEE
          	:*.  *:  .        :  ..    *.           . .  .  . . .    *:.

MG01115.1 	LTTGSCMTCGSLVRWPRDLNVFKCTICLTINDLKPLTPGET-GTGRSRDE--GGPLSHYS
NCU06756.1	YSTGNCMTCGCLVRWPKELSVFKCTICLTVNDLQPSSSGQGQGQGPRRDASRNSPPAAEE
FG06272.1 	FATGNCMTCGSLVRWPRDLKVFKCTICTTVNDLEPLGVDHD-GSKPRKDANHGPSQANTP
AN0444.1  	LTTGKCMTCNNTVRWPRHLKVFRCTECLTVNDLDPCQDSKG-SVNLSTRKNAKPIQTIPR
          	 :**.****.  ****:.*.**:** * *:***.*   ..  .       .     :   

MG01115.1 	1QP~--VSLE~HTKLLIRQCLRNFVASALAP----HANIKPNTQASLPMTIPQNGPGRPH
NCU06756.1	~RV~--FSHG1HTKSLATQCLRSYLSSALRSKSGRQADVQPDS-YFTLAKVPSQDQQESS
FG06272.1 	~RV1THVSVE~HTKRLVNQSIQSYLTKRLHPIPKPPTEPPPEPPVQSKLSFSSRMQATSR
AN0444.1  	~KA1IPLTVE~RTKNIIDRCITTYLQQCLGAPPVPPKDTSSESFRQTSSKASEHLTVPQH
          	 :    .:   :** :  :.: .:: . * . .    :  .:.      . ...      

MG01115.1 	ASTFDNENQEPSLFTPELVESPFLEAPGSFPNPSLLP--------PRSYSTSYSDQRPRM
NCU06756.1	LSSPSDPGRVPAATSRAVGVSPQPQRDGLTVPNTDLDPQQRSNRTPRSYSSSYPEKRPAI
FG06272.1 	NLSPIDPKKEATLSSKSPAIQVSHYTFDEEPKLRTNPVRTNSSPMPRSPSSSYSDRPPVQ
AN0444.1  	PTAPRG-RSVSSAGTREKADGGDTELGNFSYSKNPYP--------PVPPPKDSRNAPNSV
          	  :  .    .:  :            .                 * . ...  :     

MG01115.1 	EGLFRQQDRQGSNG--TGHELDPKKMFKPLEDYLFTCFTSYRCLQFSFGGPRGLNSQARH
NCU06756.1	PDLVLQAPGPQASPQIANPEIDSRRIFKRLEDYIISCLTSYQCLNTSFSTTRPSHQPRPS
FG06272.1 	KMLQDNVPRETRKPSLDISENDPKRIFKALEEYVVACFGSFECINSSFSTQ-PRPAMRNA
AN0444.1  	NSRERSRLRE------SGKEKHP-YIFRELEDYIISGLKGCDTLNVSFNSA--HHAPRAT
          	     .             * ..  :*: **:*:.: : .   :: **.           

MG01115.1 	MDDTPRRRTPERAQDRQPESRHVRS-----HSRSHSKVSAAAEEPIAELDAKLLLLGDFA
NCU06756.1	GEPERRRRPTQSQPQPRPQPPPIP-------QPEARKNSVTTEQPFVELDAKMLLLGDFA
FG06272.1 	SEATRRKPAPPDEPREQRGHRRVPSENHALREQRQQRTAPPVEDSTFDIDPKMLLLGDFA
AN0444.1  	PENSQPERDVERNPRR------------------------AQVDTAVELDAKTLLVGDLV
          	 :    .                                 .  :.  ::*.* **:**:.

MG01115.1 	ENGSWWTGGQDE-VPRRGSSAGIRPDGIPTAPMPATKSPGLDWSEMEEWYNTVINAAQPW
NCU06756.1	ENGLWWTGGHEEKVPGRSSSN--RSD--PGASAVSPRSPRIDWAEVEDWYTAVLDAVRSW
FG06272.1 	ENGTWWMGNQEESRPRRPSAH--RVDRSPSNATVYTKAPQLNWGDLMAWYTAIVSPAKGW
AN0444.1  	ENSSWWMVDEDSTGVPSHSQH-----KHRTTKIVSSRSPRINWIDVDKWYQLVLTAGTTW
          	**. **  ..:..     *                .::* ::* ::  **  :: .   *

MG01115.1 	KAVYDGMVAGGQ-----LSPAPEADLRRLETQILAGQDHAQRTLLKATETLLKRPGRPLT
NCU06756.1	PKFYDELVSEDS-----SLAVPPEALKEIEAQLLVGQDHVHRSLLRASETIMKRPGRLMT
FG06272.1 	FALYEEICQEQN-----FQTPPQRELQSIERELLQGQEHARRVLLKATEMLLKRPGRPLK
AN0444.1  	VEKWSVMKVSEEEDRARAEAWLMADLSVIEKEFTDSRAHLHRTLLKATESLLKRPRRPLK
          	   :. :    ... :   .     *  :* ::  .: * :* **:*:* ::*** * :.

MG01115.1 	DPTDLRFLLLILSNPLLAA------RCRKFTGHYGHSCEKERGDG-GSSWSDVPRGTGPA
NCU06756.1	APHELRFILILAANPLLHA------NYKPYTGEFRHMDS-------FWSTQG-----GPS
FG06272.1 	DPTDLRFLLVIIENPLLHD------NENLFRGIIQPENNSPSGANFARSRKGSVPETGPL
AN0444.1  	KPDDIRFLLILLYNPLIYPPSSTLANLTVVDGELKPSHSVGNSSEGRAAGNKAHTAKAQS
          	 * ::**:*::  ***:  .::: :.     *      .   .     : .     ..  

MG01115.1 	SG--QHSGIIKRILGLMSNSSNDCHNQLVAWLARFPESRFTDFKDFVSGFMAYRLNRQNE
NCU06756.1	SG--RHSGIIKRIVGLLSNTPNECHNHLVNWFARYPEPLFIKTKDLISSFLAFRLNRQNE
FG06272.1 	SG--QHSGIIKRIVGLISNSSPECHNQLISWFARYHPFRFIRTKEIASGFLTYRMIRQSG
AN0444.1  	GGPVQHPGIIKRILGLLSDLPSDCHHYLVSWFSRFSVGHFEKLVELVGRFVTYRLSRQHV
          	.*. :*.******:**:*: . :**: *: *::*:    *    :: . *:::*: **  

MG01115.1 	KKHDVKFDVTDGLIPIVS--AGRSAATLHAALGPSSGSAKKQKEKPKKTIVYDDWQVKAA
NCU06756.1	KKYDARVDITAGLIPSLN--AGRSAASLHAALGSSQ-ASGKKQK-EKKKIYNEDWQIKAS
FG06272.1 	KKQEVKVDITAGLIPQMQ--EGRSGAYLYDEINRST-SSKKAKEPEKKIMYGEDWQIRAS
AN0444.1  	RRKSESANDDNYLIPSLSSAAMNTPSELHAAINGINQNKQAKKKADAPMTYSDDWQVRAA
          	:: .   :    *** :.::  .: : *:  :.         ::        :***::*:

MG01115.1 	ARVMTLIFSANNTHYSRRNPQDQGFSHLNADSMRAKGRVHARGQLLPTSDFYMALLDNS-
NCU06756.1	AQVLALIFAANNTGHSRRSLANLTDGPV----ASTRDRVQMRGQILATSDFYTTLLDDS-
FG06272.1 	SRVLALLFTANNLPHNRK--NDETSSPS----RERCTGAHAHGQILPTSDFYNSMIDYT-
AN0444.1  	GRVMALLFTANNADIPRKPPGTESAVMS-------RQQGAHRGHIIPISSFYNTRLDYSN
          	.:*::*:*:***    *:                       :*:::. *.** : :* :.

MG01115.1 	ELTQDFEAWETKRAKFSFCQYPFLLSVWAKIQILEHDAKREMDKKARDAFFNSIMSRRSF
NCU06756.1	DLVADFETWESKKGKFAFCQFPFLLSIGAKIQILEYDAKRQMEDKARDAFFNSILTHRVI
FG06272.1 	DLVADFENWESRKSKFTFCQYPFLLSIWAKTHILEHDARRQMQSKARDAFFDSIMSRKAI
AN0444.1  	LVVADFEAWESRTTKFSFCQYPFFLSIGAKIQILEHDARRQMEVKAREAFFSSILTRKAV
          	 :. *** **::  **:***:**:**: ** :***:**:*:*: ***:***.**:::: .

MG01115.1 	DQYLYLNVRRDCLVEDSLKAVSEVIGSGGEDIKKGLRITFRGEEGVDAGGLRKEWFLLLV
NCU06756.1	QQHLVLDVRRDCLVDDSLKAVSEVIGSGGEDIKKGLKINFKGEEGIDAGGLRKEWFLLLV
FG06272.1 	NQFLELNVRRDCLVDDSLKAVSEVIGSGSEDIKKGLRITFNGEEGVDAGGLRKEWFLLLV
AN0444.1  	SQYFVLRVRRECLVEDSLQRVSEVLGSSPEEMKKGLRIGFVGEEGVDAGGLRKEWFLLLV
          	.*.: * ***:***:***: ****:**. *::****:* * ****:**************

MG01115.1 	REVFNPDHG1LFVYDDDSHYCYFNPNSFETSDQYFLVGVVFGLAIYNSTILDVAFPPFLF
NCU06756.1	REVFNPDHG1MFVYDEDSQYCYFNPASLEPSEQYFLVGVVFGLAIYNSTILDVALPPFAF
FG06272.1 	REVFNPDHG1LFLYDEDSQYCYFNPNAFETSDQFFLIGVVMGLAIYNSTILDVALPPFAF
AN0444.1  	REVFDPHHG1LFIYDEDSRYCYFNPYCLESSEQFFLVGVVLGLAIYNSIILDIALPPFAF
          	****:*.** :*:**:**:****** .:*.*:*:**:***:******* ***:*:*** *

MG01115.1 	RKLLAASPQPSGASPAHSRPPMTYTLEDLAEFHPMLAKGLRQLLEFEGDVEETFCLDFVV
NCU06756.1	RKLLMAAPPP-TLATSQPRQPMTYSLDDLAEYRPRLASGLRQLLEYDGDVESTFCLDFVV
FG06272.1 	RKLIASAPTHGTGASAHPKPPMRYTLEDLAEYRPRLARGLRQLLEYEGNVEETFCLDFVV
AN0444.1  	KKLLAGAPQTTGPQPLTTRPTYKCSLEDLAEYRPVLARGLRALLEFEGDVAETFCHTFVA
          	:**: .:*      .  .: .   :*:****::* ** *** ***::*:* .***  **.

MG01115.1 	DVPKYGAIERVPLCQGGERRAVTNANRREYVDLHVRYILDTAVARQFDPFKRGFFTVCGG
NCU06756.1	DIERYGSTERVSLCPNGERRPVTNANRREYVDLYVRYLLDTAVTRQFEPFKRGFYTVCGG
FG06272.1 	DMDKYGTQVQVPLCRGGERIPVTNSNRREYVDLYVRHVIDVSVTRQFEPFKRGFYTVCGG
AN0444.1  	DVDRYGEVVSVPLCPGGEKKPVTNANRREFVDLFVHYHLDTAVARQFEPFRRGFFSVCGG
          	*: :**    *.** .**: .***:****:***.*:: :*.:*:***:**:***::****

MG01115.1 	NALSLFRPEEIELLVRGSEETLDVVSLKSAAEYDGWMRNNKKRPEESSPSGGDGTEVAEE
NCU06756.1	NALSLFRPEEIELLVRGSDESLDISALKSAATYDNWSTKNPVETE---------------
FG06272.1 	NALSLFRPEEIELLVRGSDEELDINSLRGVAEYDNWGNKKSDGSE---------------
AN0444.1  	NALSLFRPEEIELLVRGSDEQLDVKSLRAVATYLNWGTPKPESVP---------------
          	******************:* **: :*:..* * .*   :                    

MG01115.1 	VPTLRWFWDSFQRASPEDQRKLLIFITGSDRIPAMGAASLSIKILCLGDDCDR~YPTART
NCU06756.1	-PTVGWFWELFEEASPADQRKLLLFITGSDRIPAGGAAALSIRIACLGEDCGR~YPTART
FG06272.1 	-PVIDWFWETFQEATSQDQRKLLSFITGSDRIPAMGAAVLPIKISCLGEDEGR2K-IGED
AN0444.1  	--VVRWFWEYFERANPEAQRKILSFVTSSDRIPAMGATSLNIQLICLGDDSPR~FPTAHT
          	  .: ***: *:.*..  ***:* *:*.****** **: * *:: ***:*  *  . .. 

MG01115.1 	CFNTLALWRYESKEKLERMLWGAVFESEGFGLK
NCU06756.1	CFNSLALWKYGSRERLKEVLWTAVLESEGFGLK
FG06272.1 	VMDCCA---------------------------
AN0444.1  	CFNRLGLYRYKTREKFERLLSEAVLNSEGFGLK
          	 ::  .     : .  .     :  .:.. . .
```
